# Use of e-Cigarettes in Cigarette Smoking Cessation: Secondary Analysis of a Randomized Controlled Trial

**DOI:** 10.2196/48896

**Published:** 2023-11-09

**Authors:** Margarita Santiago-Torres, Kristin E Mull, Brianna M Sullivan, Jonathan B Bricker

**Affiliations:** 1 Fred Hutchinson Cancer Center Seattle, WA United States; 2 Department of Psychology University of Washington Seattle, WA United States

**Keywords:** acceptance and commitment therapy, cigarette smoking, digital behavioral interventions, e-cigarettes, smoking cessation, smartphone apps, vaping, mobile phone

## Abstract

**Background:**

Many adults use e-cigarettes to help them quit cigarette smoking. However, the impact of self-selected use of e-cigarettes on cigarette smoking cessation, particularly when concurrently receiving app-based behavioral interventions, remains unexplored.

**Objective:**

This study used data from a randomized trial of 2 smartphone apps to compare 12-month cigarette smoking cessation rates between participants who used e-cigarettes on their own (ie, adopters: n=465) versus those who did not (ie, nonadopters: n=1097).

**Methods:**

The study population included all participants who did not use e-cigarettes at baseline. “Adopters” were those who self-reported the use of e-cigarettes at either 3- or 6-month follow-ups. “Nonadopters” were those who self-reported no use of e-cigarettes at either follow-up time point. The primary cessation outcome was self-reported, complete-case, 30-day point prevalence abstinence from cigarette smoking at 12 months. Secondary outcomes were missing-as-smoking and multiple imputation analyses of the primary outcome, prolonged abstinence, and cessation of all nicotine and tobacco products at 12 months. In logistic regression models, we first examined the potential interaction between e-cigarette use and treatment arm (iCanQuit vs QuitGuide) on the primary cessation outcome. Subsequently, we compared 12-month cigarette smoking cessation rates between adopters and nonadopters separately for each app.

**Results:**

There was suggestive evidence for an interaction between e-cigarette use and treatment arm on cessation (*P*=.05). In the iCanQuit arm, 12-month cigarette smoking cessation rates were significantly lower among e-cigarette adopters compared with nonadopters (41/193, 21.2% vs 184/527, 34.9%; *P*=.003; odds ratio 0.55, 95% CI 0.37-0.81). In contrast, in the QuitGuide arm, 12-month cigarette smoking cessation rates did not differ between adopters and nonadopters (46/246, 18.7% vs 104/522, 19.9%; *P*=.64; odds ratio 0.91, 95% CI 0.62-1.35).

**Conclusions:**

The use of e-cigarettes while concurrently receiving an app-based smoking cessation intervention was associated with either a lower or an unimproved likelihood of quitting cigarette smoking compared to no use. Future behavioral treatments for cigarette smoking cessation should consider including information on the potential consequences of e-cigarette use.

**Trial Registration:**

ClinicalTrials.gov NCT02724462; https://clinicaltrials.gov/study/NCT02724462

## Introduction

### Background

Electronic nicotine delivery systems or vaping (referred to hereafter as “e-cigarettes”) are battery-powered devices that simulate the experience of smoking by heating a liquid solution into an aerosol, which is then inhaled by the user [1]. This makes them a popular alternative for individuals seeking a less harmful nicotine delivery method. One of the key distinctions is that e-cigarettes operate without combustion, resulting in significantly lower levels of carcinogens and harmful compounds compared to the smoke produced by traditional cigarettes [2-4]. While e-cigarettes offer the potential to substantially reduce exposure to the harmful substances found in cigarette smoke [5,6], it is also important to acknowledge that e-cigarettes are not risk free. The long-term effects of vaping are currently under investigation, and concerns have been raised particularly regarding nonsmokers and young individuals who may take up vaping [7-9].

According to a survey conducted in the United States (US) by the Centers for Disease Control and Prevention in 2020, among adult e-cigarette users, 34.7% reported using e-cigarettes to quit smoking cigarettes, and 29.8% reported using e-cigarettes to reduce their cigarette smoking [1]. While there is significant interest among health providers, regulators, and treatment-seeking individuals alike regarding the impact of e-cigarettes on cigarette smoking cessation [10], the current evidence on the benefits and harms of using e-cigarettes for cigarette smoking cessation is mixed. For example, observational studies using data from the US Population Assessment of Tobacco and Health cohort suggest that e-cigarette use facilitates cigarette smoking cessation in real-world settings [11-13]. Moreover, a recent Cochrane review of e-cigarette randomized controlled trials (RCTs) for smoking cessation found evidence, albeit of very low certainty, of higher quit rates in participants using e-cigarettes compared with behavioral support alone or no support [14]. However, it is important to note that the authors of the review highlighted the very low certainty of these results due to imprecision and potential bias. In contrast, results from 2 prospective studies of RCTs among treatment-seeking individuals who used e-cigarettes while concurrently receiving behavioral cessation treatments suggest that e-cigarette use impedes cigarette smoking cessation [15,16]. Thus, it remains unclear whether e-cigarette use aids or hinders smoking cessation among treatment-seeking individuals while concurrently receiving behavioral support.

In-person traditional behavioral interventions such as counseling, support groups, and pharmacotherapy (eg, nicotine replacement therapy or medications to aid cessation) have been used to aid in cigarette smoking cessation [17]. However, the widespread use of smartphones and other digital devices has opened new avenues for delivering behavioral interventions through digital therapeutics [18], potentially enhancing the efficacy and accessibility of cessation treatments. The impact of e-cigarettes on the efficacy of digital interventions for smoking cessation is completely unexplored. With hundreds of smartphone apps designed for smoking cessation currently available, and given that 85% of US adults own smartphones, these apps are highly accessible [19,20]. In the US alone, smoking cessation apps are downloaded around 2 million times per year, highlighting their popularity and potential impact (R Nelson; SensorTower.com; personal communication; April 15, 2020). App-based interventions are particularly relevant because they can provide access to evidence-based cessation treatments that may otherwise be limited. Due to their low cost and potential for high population reach, app-delivered interventions could be an effective solution for many people seeking to quit smoking.

Among smoking cessation apps, iCanQuit is the only app that has demonstrated efficacy for cigarette smoking cessation in a full-scale RCT with long-term follow-up [21]. The iCanQuit app is based on acceptance and commitment therapy (ACT) principles [22] for behavior change, which teach users skills to accept their cravings to smoke and allow them to pass without smoking [23-25]. Our group developed and tested the iCanQuit app against the QuitGuide app. QuitGuide was selected because it follows standard behavioral approaches recommended by the US Clinical Practice Guidelines (USCPG) [21]; it is a smartphone app and thus avoids confounding treatment content with delivery modality; and it is nonproprietary and freely available to the public, providing maximal transparency and replicability.

The results from the iCanQuit parent trial showed (1) potential for high reach nationwide, (2) higher efficacy for cigarette smoking cessation of iCanQuit relative to QuitGuide (12-month quit smoking rates: 28% in iCanQuit vs 21% in QuitGuide; *P*<.001), and (3) higher engagement with iCanQuit compared to QuitGuide. Although the iCanQuit app has shown promising results in helping individuals quit smoking, it is unknown to what extent concurrent use of e-cigarettes might impact their cigarette smoking cessation rates. Understanding the impact of e-cigarettes on the efficacy of app-based cessation interventions is crucial as it can inform the development of content for users who intend to combine e-cigarettes with app-based interventions.

### Objectives

To help address this unexplored question, this study aimed to compare cigarette smoking cessation rates between individuals who adopted e-cigarettes on their own and those who did not, within the context of app-based smoking cessation interventions. We hypothesized that the use of e-cigarettes would be associated with a greater likelihood of quitting cigarette smoking compared with no use. This hypothesis is based on the role of nicotine-containing e-cigarettes in alleviating withdrawal symptoms associated with dependence, thereby enhancing abstinence. We further hypothesized that e-cigarette adopters within the iCanQuit arm would have higher smoking cessation rates than those in the QuitGuide arm. This hypothesis was grounded in the evidence demonstrating a greater efficacy of iCanQuit for smoking cessation compared to QuitGuide [21].

To test these hypotheses, the potential interaction between the use of e-cigarette and treatment arm was first investigated (iCanQuit vs QuitGuide) on the primary cessation outcome. Subsequently, 12-month rates of cigarette smoking cessation were compared between adopters and nonadopters separately for each app. Finally, to gain a deeper understanding of the potential mechanisms underlying the impact of e-cigarettes on combustible cigarette smoking cessation, we conducted post hoc exploratory analyses to compare the following aspects between adopters and nonadopters: (1) self-selected use of cessation pharmacotherapy, (2) rates of cigarette smoking cessation for participants indicating they adopted e-cigarettes specifically as a cessation aid versus those who did not [26,27], (3) app engagement, and (4) number of quit attempts [28,29]. Moreover, we examined the associations between (5) the number of quit attempts and (6) levels of nicotine dependence with rates of cigarette smoking cessation at 12 months.

## Methods

### Overview

Data from this secondary analysis were from participants (aged 18 years or older) in the iCanQuit parent RCT who reported no e-cigarette use at baseline. Details of the iCanQuit RCT have been previously published [21]. Briefly, the iCanQuit RCT enrolled a racially or ethnically diverse sample of 2415 adults who smoke combustible cigarettes to participate in a 12-month app-delivered cigarette smoking cessation intervention. The primary aim of the trial was to test the efficacy of the ACT-based iCanQuit app against the USCPG-based QuitGuide app for cigarette smoking cessation at the 12-month follow-up.

### Ethical Considerations

Study procedures were approved by the Fred Hutchinson Cancer Center Institutional Review Board (reference number: 8317). All participants were provided with information about the study and signed informed consent forms before participation. This study is registered in the ClinicalTrials.gov (NCT02724462).

### Eligibility Criteria

Eligibility criteria included daily cigarette smoking, smartphone access, wanting to quit combustible cigarette smoking within the next 30 days, and wanting to quit within the next 30 days any other concurrently used nicotine or tobacco products. Exclusion criteria included being unable to read English, receiving smoking cessation treatment, having used either iCanQuit or QuitGuide in the past, or having a household member already enrolled in the study. Any use of e-cigarettes or other noncigarette nicotine or tobacco products was not an exclusion criterion, and data on their use were collected at each data collection time point. The approved study protocol was published alongside the main outcomes paper and is readily accessible for review [21].

### Recruitment and Enrollment

Facebook ads were the primary source of recruitment for all trial participants (1281/1562, 82%), followed by a survey sampling company (203/1562, 13%), friends or family (47/1562, 3%), and search engine results (16/1562, 1%). The period of recruitment of all trial participants was May 2017 through September 2018. Participants completed a web-based screening survey and were notified of their eligibility via email. They then clicked on a secured emailed link to the study website, where they provided consent and completed the baseline survey. Consent to participate included collecting app use data via Google Analytics (number of logins, time spent using the app, and specific features used). Activation of the assigned app on the participant’s smartphone was conducted by entering the login code sent to them in their trial enrollment email.

At each enrollment step, the study was presented as a comparison of 2 smartphone apps for cigarette smoking cessation. After completing the baseline survey, participants were then randomized 1:1 to receive iCanQuit or QuitGuide, both of which were accessible for 12 months. Randomization by permuted blocks of size 2, 4, and 6 was stratified by positive screening for depression (Center for Epidemiological Studies Depression Scale-20 score ≤15 vs ≥16), daily smoking frequency (≤20 vs ≥21 cigarettes per day), minoritized race or ethnicity backgrounds, and education level (less than high school vs some college or higher education). Neither research staff nor study participants had access to upcoming randomized study group assignments. For blinding, each app was branded as “iCanQuit” and did not mention either ACT or QuitGuide. Contamination between apps was avoided with a unique username and password provided only to the individual user and by having an eligibility criterion of not having other household members participating in the study.

Data collection occurred between August 2017 through December 2019 via web-based study surveys at the 3-, 6-, and 12-month follow-ups. For trial integrity, the follow-up data were collected by the survey research unit that was blinded to random assignments, and cessation outcomes data were collected outside of the intervention apps. Participants were compensated for completed data collection. For each follow-up time point, participants received US $25 for completing the follow-up survey. Participants would receive an additional US $10 bonus if the encrypted web-based survey was completed within 24 hours of the initial email invitation to complete the survey, with up to US $105 in total compensation per participant.

### Study Population

The data for this secondary analysis included a subsample of trial participants from the iCanQuit parent RCT who had available data on e-cigarette use after randomization. This subset comprised 1562 participants, which accounted for 64.7% (1562/2415) of the total sample. Within this subsample, we categorized participants into two distinct groups based on their self-reported use of e-cigarettes after randomization: (1) “adopters,” defined as individuals who reported no e-cigarette use in the past 30 days at baseline and reported using e-cigarettes within the past 30 days at either the 3- or 6-month follow-ups (465 adopters); and (2) “nonadopters,” defined as individuals who reported no e-cigarette use in the past 30 days at baseline, 3-month, and 6-month follow-ups (1097 nonadopters). Participants who reported using e-cigarettes at baseline (n=575) were excluded from the analysis, as were those with missing e-cigarette use data (n=278). It is important to emphasize that the use of e-cigarettes during the 12-month intervention period was entirely self-selected by the participants.

### Smartphone App–Based Interventions

#### iCanQuit

Details of the iCanQuit app have been previously published [21]. Briefly, participants who had access to the ACT-based iCanQuit app for 12 months received 8 levels of intervention content based on 2 key processes of ACT: acceptance of cravings to smoke and enactment of core life values that motivate living a smoke-free life. After setting up a personalized quit plan, users are taken to the home screen, where they can progress through 8 levels of the intervention content. The program is self-paced, and content is unlocked in a sequential manner. In the “Preparing to Quit” phase, iCanQuit focuses on helping the user develop acceptance of physical sensations, emotions, and thoughts that trigger smoking and allowing these triggers to pass without smoking via mindfulness and perspective-taking. There is an “Urge Help” feature that is tailored to the type of trigger experienced by the user as well as a tracking feature that encourages participants to track the number of cigarettes smoked and urges passed. In the “After You Quit” phase, iCanQuit focuses on helping the user stay motivated and preventing relapse.

#### QuitGuide

Details of the QuitGuide app have been previously published [21]. Briefly, the QuitGuide app developed by the National Cancer Institute is based on the USCPG for smoking cessation [30]. QuitGuide contained 4 sections of content. “Thinking About Quitting” focuses on motivations to quit by encouraging users to think of reasons for quitting and providing information on the general health consequences of smoking and quitting. “Preparing to Quit” helps users develop a quit plan; identify smoking behaviors, triggers, and reasons for being smoke-free; and identify social support for quitting. “Quitting” teaches skills for avoiding cravings to smoke, such as finding replacement behaviors (eg, chewing on carrot sticks) and staying busy. “Staying Quit” presents tips, motivations, and actions to stay smoke-free and skills for coping with cravings and trying to be positive.

#### Major Similarities Between iCanQuit and QuitGuide

Both apps provide education and skills for preparing to quit cigarette smoking and for preventing relapse after quitting; actionable plans for quitting cigarette smoking; skills for coping with cravings to smoke; and education on common triggers to smoke, barriers to cessation, and how to seek support for smoking cessation. Both apps also provided education on US Food and Drug Administration–approved medications for smoking cessation. However, neither app provided e-cigarettes as part of the behavioral interventions, encouraged their use, or provided information on the potential risks or benefits of using e-cigarettes to quit combustible cigarette smoking. The details on the features and functionalities of the apps as well as a comparison have been previously published [21].

### Assessments

#### Baseline Survey

The baseline survey collected information on sociodemographic factors such as age, gender, race, education level, and household income. In addition, participants were screened for depression using the Center for Epidemiological Studies Depression Scale-20 and panic disorder and posttraumatic stress disorder (PTSD) using self-report measures [31-33]. Questions related to smoking behavior were also included, such as the number of combustible cigarettes smoked per day, smoking history (years of smoking and past quit attempts), use of e-cigarettes in the past month, and whether family and friends also smoked. The Fagerström Test for Cigarette Dependence (FTCD) [34] was used to assess the level of nicotine dependence. Participants were also asked about their confidence in quitting cigarette smoking and alcohol use.

#### Smoking Cessation

Study questionnaires at the 3-, 6-, and 12-month follow-ups asked participants whether they abstained from combustible cigarette smoking for the past 30 days and the date of their last cigarette. Consistent with the parent trial, the primary smoking cessation outcome was self-reported, complete-case, and 30-day point prevalence abstinence (PPA) from cigarette smoking at 12 months. Secondary outcomes at 12 months included 30-day PPA missing-as-smoking and multiple imputation; 30-day PPA from all nicotine and tobacco products (ie, e-cigarettes, chewing tobacco, snus, hookahs, cigars, cigarillos, tobacco pipes, and kreteks); prolonged abstinence (between 3 and 12 months); and harm reduction, defined as a substantial reduction (≥50%) in average daily cigarette consumption between baseline and 12 months [35,36].

#### Self-Selected Use of e-Cigarettes and Cessation Pharmacotherapy

Participants were asked via web-based study questionnaires at baseline and 3-, 6-, and 12-month follow-ups: “In the last 30 days, how often did you use any kind of e-cigarette or vaping?” Response options included (1) not at all, (2) less than once a month, (3) once a month or more but less than once a week, (4) once a week or more but not daily, and (5) at least daily. To assess self-selected use of cessation pharmacotherapy, participants were asked: “Since the date you joined the study, did you ever use any of the following nicotine replacement therapies or medications to help you quit smoking?” Response options included (1) nicotine gum, (2) nicotine patches, (3) e-cigarettes, (4) Chantix, (5) Zyban or Wellbutrin, or (6) “free text.” The response to this question was also used to assess self-selected use of cessation pharmacotherapy and whether e-cigarettes were intentionally used to aid quitting.

#### Engagement With the App-Based Interventions

The engagement was measured via Google Analytics and included the number of times users interacted with their assigned app (ie, number of logins) by 6 months after randomization.

#### Quit Attempts

A number of quit attempts were assessed at each time point using the survey question, “About how many times since the start of the study did you quit smoking for at least 24 hours?

### Statistical Analysis

Sociodemographic characteristics and smoking behaviors of participants were compared between adopters and nonadopters using 2-tailed *t* tests for continuous variables and chi-square tests for categorical variables. To test whether there was an interaction between adopting e-cigarettes and app assignment (iCanQuit vs QuitGuide) on the primary cessation outcome, we evaluated the interaction term in a logistic regression model. To compare 12-month cigarette smoking cessation rates between adopters and nonadopters, logistic regression models were used. For the multiple imputation of missing 30-day PPA from cigarette smoking, multivariate imputation by chained equations in the R package *mice* [37] was used to create 10 complete data sets and pool logistic regression model results [38]. Differences in app engagement and the number of quit attempts between adopters and nonadopters were explored using negative binomial models in the R package *MASS* [39]. We used logistic regression models to explore whether the adoption of e-cigarettes as an aid to cessation impacted smoking cessation. All cessation models were adjusted for factors used in stratified randomization to avoid losing power and obtaining an incorrect 95% CI [40]. These baseline factors included positive screening for depression, daily smoking frequency, education, and minority race or ethnicity backgrounds. We also adjusted for baseline characteristics that both differed between e-cigarette adopters and nonadopters and were associated with the cessation outcome to reduce the potential for confounding [41,42]. The R software (version 4.2.3; R Foundation for Statistical Computing) was used for all statistical analyses, and all statistical tests were 2-sided with α=.05 [43].

## Results

### Participants Characteristics

Participants included in this analysis (ie, adopters and nonadopters combined) were on average 38.9 years old, 72.9% (1139/1562) female, and 32.8% (512/1562) were from minoritized racial groups (American Indian or Alaska Native, Asian, Black or African American, Native Hawaiian or Pacific Islander, or multiple races; Table 1). A substantial proportion (657/1562, 42.1%) reported having a high school diploma or less education; 47.3% (739/1562) were unemployed, disabled, or out of the labor force; and 37.3% (583/1562) reported household incomes of US $20,000 per year or less. Almost half (711/1553, 45.8%) screened positive for depression, 26.1% (401/1537) screened positive for panic disorder, and 41.7% (647/1550) screened positive for PTSD. The FTCD measure of nicotine dependence was mean 5.9 (SD 2.0), indicating moderate to high nicotine dependence, and an overwhelming majority (1323/1562, 84.7%) reported smoking for ≥10 years.

**Table 1:** Baseline characteristics between nonadopters and adopters.

Characteristic			All participants (N=1562)		Nonadopters (n=1097)		Adopters (n=465)		*P* value
Age (years; N=1562), mean (SD)			38.9 (10.9)		39.3 (10.9)		37.7 (10.8)		.008
Female (N=1562), n (%)			1139 (72.9)		809 (73.7)		330 (71)		.26
**Race** **(N=1562),** **n (%)**									.003
	American Indian or Alaska Native	39 (2.5)		31 (2.8)		8 (1.7)			
	Asian	3 (0.2)		2 (0.2)		1 (0.2)			
	Black or African American	341 (21.8)		266 (24.2)		75 (16.1)			
	Native Hawaiian or Pacific Islander	2 (0.1)		0 (0)		2 (0.4)			
	White	1050 (67.2)		713 (65)		337 (72.5)			
	Multiracial	103 (6.6)		70 (6.4)		33 (7.1)			
Hispanic or Latino ethnicity (N=1562), n (%)			130 (8.3)		83 (7.6)		47 (10.1)		.10
**Education (N=1562), n (%)**									.11
	Less than GED^a^ or high school	657 (42.1)		452 (41.2)		205 (44.1)			
	Some college, no degree	581 (37.2)		402 (36.6)		179 (38.5)			
	College degree or higher	324 (20.7)		243 (22.2)		81 (17.4)			
**Employment** **(N=1562),** **n (%)**									.30
	Employed	823 (52.7)		573 (52.2)		250 (53.8)			
	Unemployed	192 (12.3)		141 (12.9)		51 (11)			
	Disabled	242 (15.5)		178 (16.2)		64 (13.8)			
	Out of labor force	305 (19.5)		205 (18.7)		100 (21.5)			
**Income (US $; N=1562), n (%)**									.94
	<$20,000 per year	583 (37.3)		412 (37.6)		171 (36.8)			
	$20,000-$54,999 per year	715 (45.8)		499 (45.5)		216 (46.5)			
	≥$55,000 per year	264 (16.9)		186 (17)		78 (16.8)			
Married (N=1562), n (%)			503 (32.2)		338 (30.8)		165 (35.5)		.07
LGBT^b^ (N=1562), n (%)			251 (16.1)		166 (15.1)		85 (18.3)		.12
Rural residence (N=1562), n (%)			361 (23.1)		262 (23.9)		99 (21.3)		.27
**Mental health positive screening results, n (%)**									
	Depression (n=1553)^c^	711 (45.8)		478 (43.9)		233 (50.2)		.02	
	Panic disorder (n=1537)^d^	401 (26.1)		278 (25.8)		123 (26.7)		.71	
	PTSD (n=1550)^e,f^	647 (41.7)		435 (39.9)		212 (46)		.03	
**Alcohol use** **(n=1518)**									
	Drinks per day, mean (SD)	1.8 (3.6)		1.8 (3.5)		1.8 (3.9)		.96	
	Heavy drinker, n (%)^g^	216 (14.2)		157 (14.7)		59 (13.1)		.43	
**Smoking behaviors**									
	Cigarettes smoked per day (n=1518), mean (SD)	19.2 (14.4)		18.9 (13.9)		19.9 (15.3)		.17	
	FTCD^h^ score (n=1518), mean (SD)	5.9 (2.0)		5.8 (2.1)		6.0 (1.9)		.02	
	Cigarette within 5 minutes of waking (n=1518), n (%)	849 (54.4)		574 (52.3)		275 (59.1)		.01	
	Smokes greater than one-half pack per day (n=1518), n (%)	1174 (75.2)		816 (74.4)		358 (77)		.28	
	Smokes >1 pack per day (n=1518), n (%)	313 (20)		211 (19.2)		102 (21.9)		.22	
	Smoked for ≥10 years (n=1518), n (%)	1323 (84.7)		929 (84.7)		394 (84.7)		.98	
	Past 12-month quit attempts (n=1496), mean (SD)	1.4 (6.6)		1.3 (3.7)		1.7 (10.7)		.26	
	Confidence to quit (n=1518), mean (SD)^i^	64.2 (26.8)		64.5 (26.9)		63.4 (26.5)		.45	
	Close friends who smoke (n=1518), mean (SD)	2.6 (1.7)		2.6 (1.8)		2.7 (1.7)		.10	
	No. housemates who smoke (n=1518), mean (SD)	1.5 (0.9)		1.4 (0.8)		1.5 (1.0)		.03	
	Living with partner who smokes (n=1518), n (%)	567 (36.3)		384 (35)		183 (39.4)		.10	

^a^GED: General Education Development.

^b^LGBT: lesbian, gay, bisexual, or transgender.

^c^Positive screening results for depression via the Center for Epidemiological Studies Depression Scale (cutoff ≥16).

^d^Positive screening results for panic disorder via the 5-item Autonomic Nervous System Questionnaire (reporting ≥1 panic attack or worry about a recurrence within the past month indicates a positive screen).

^e^PTSD: posttraumatic stress disorder.

^f^Positive screening results for PTSD via the PTSD Checklist (scores of ≥14 indicate a positive screen).

^g^Heavy drinking is defined as 4 or more drinks on a typical drinking day for women and 5 or more drinks on a typical drinking day for men within the past 30 days.

^h^FTCD: Fagerström Test for Cigarette Dependence.

^i^Range 0-100, where 0 indicates not at all confident and 100 indicates extremely confident.

Overall, 29.8% (465/1562) of participants adopted e-cigarette use between baseline and the 6-month follow-up, while 70.2% (1097/1562) reported no use of e-cigarettes between baseline and the 6-month follow-up. A descriptively higher, but nonsignificantly different, proportion of QuitGuide participants adopted e-cigarettes compared with iCanQuit participants (QuitGuide: 254/797, 31.9% and iCanQuit: 211/765, 27.6%; *P*=.17).

Adopters were younger, more likely to be White, and screened positive for depression and PTSD compared with nonadopters. Adopters were also more dependent on nicotine than nonadopters (FTCD score: mean 6.0, SD 1.9 for adopters vs mean 5.8, SD 2.1 for nonadopters) and more likely to report having their first cigarette within 5 minutes of waking and to live with other people who smoke. Outcome data retention rates were high and did not differ between adopters and nonadopters (439/465, 94.4% vs 1049/1097, 95.6%; *P*=.32).

### Interaction Effect of Adopting e-Cigarettes and Treatment Arm on Smoking Cessation

There was suggestive evidence (*P*=.05) that the association between adopting e-cigarettes and 12-month combustible cigarette smoking cessation was moderated by treatment arm assignment (iCanQuit vs QuitGuide). Therefore, the comparison between cigarette smoking cessation rates between adopters and nonadopters was conducted separately for each arm.

### Smoking Cessation Rates Between Adopters and Nonadopters and by Treatment Arm

Specifically, results showed that 12-month smoking cessation rates in the iCanQuit arm were lower among adopters as compared to nonadopters (41/193, 21.2% vs 184/527, 34.9%; *P*=.003; odds ratio [OR] 0.55, 95% CI 0.37-0.81; Table 2). Results were similar and statistically significant for the secondary outcomes: missing-as-smoking imputation (41/211, 19.4% vs 184/554, 33.2%; *P*=.002), multiple imputation (461/2110, 21.8% vs 1909/5540, 34.5%; *P*=.01), and 30-day PPA from all nicotine and tobacco products (30/193, 15.5% vs 167/526, 31.7%; *P*<.001). Furthermore, prolonged abstinence from cigarette smoking between 3- and 12-month follow-ups was about 3 times lower among adopters as compared with nonadopters (7/148, 4.7% vs 75/434, 17.3%; *P*=.001; OR 0.26, 95% CI 0.11-0.57). Finally, there was no difference in daily cigarette reduction rates (ie, reduced by at least half) between adopters and nonadopters (*P*=.99).

**Table 2:** Smoking cessation rates at 12 months between adopters and nonadopters and by arm^a^.

Cessation outcomes and treatment arm				Nonadopters, n/N (%)		Adopters, n/N (%)		OR^b^ (95% CI)	*P* value
**30-day PPA^c^ from cigarette smoking**									
	**Complete case^d^**								
		iCanQuit	184/527 (34.9)		41/193 (21.2)		0.55 (0.37-0.81)		.003
		QuitGuide	104/522 (19.9)		46/246 (18.7)		0.91 (0.62-1.35)		.64
	**Missing-as-smoking^d^**								
		iCanQuit	184/554 (33.2)		41/211 (19.4)		0.53 (0.36-0.79)		.002
		QuitGuide	104/543 (19.2)		46/254 (18.1)		0.93 (0.63-1.37)		.70
	**Multiple imputation^d^**								
		iCanQuit	1909/5540 (34.5)		461/2110 (21.8)		0.57 (0.38-0.85)		.01
		QuitGuide	1078/5430 (19.9)		471/2540 (18.5)		0.91 (0.62-1.34)		.63
**30-day PPA from nicotine and tobacco products**									
	iCanQuit			167/526 (31.7)		30/193 (15.5)		0.42 (0.27-0.65)	<.001
	QuitGuide			91/522 (17.4)		31/246 (12.6)		0.68 (0.44-1.06)	.09
**Prolonged cigarette abstinence^d,e^**									
	iCanQuit			75/434 (17.3)		7/148 (4.7)		0.26 (0.11-0.57)	.001
	QuitGuide			34/435 (7.8)		6/187 (3.2)		0.37 (0.15-0.91)	.03
**Reduction in the number of cigarettes smoked by ≥50%^f,g^**									
	iCanQuit			65/207 (31.4)		29/90 (32.2)		1.00 (0.58-1.72)	.99
	QuitGuide			84/268 (31.3)		32/112 (28.6)		0.85 (0.51-1.44)	.55

^a^All models were adjusted for factors used in stratified randomization including positive screening for depression, daily smoking frequency, education, and minority race or ethnicity backgrounds.

^b^OR: odds ratio.

^c^PPA: point prevalence abstinence.

^d^Additional covariate is posttraumatic stress disorder positive screening.

^e^Prolonged abstinence is defined as no smoking since 3-month after randomization, using self-reported date of last cigarette.

^f^Reduction in the number of cigarettes smoked from baseline to 12 months defined as 50% reduction or more.

^g^Additional covariates are age, posttraumatic stress disorder positive screening, and smoking first cigarette within 5 minutes of waking.

In the QuitGuide arm, adopting e-cigarettes was not associated with 12-month combustible cigarette smoking cessation (46/246, 18.7% adopters vs 104/522, 19.9% nonadopters; *P*=.64; OR 0.91, 95% CI 0.62-1.35) with similar results for the secondary outcomes of missing-as-smoking and multiple imputation. There were no significant differences between adopters and nonadopters in 30-day PPA from all nicotine or tobacco products or in daily cigarette reduction. However, the odds of prolonged abstinence from cigarette smoking were roughly 3 times lower among adopters as compared with nonadopters (6/187, 3.2% vs 34/435, 7.8%; *P*=.03; OR 0.37, 95% CI 0.15-0.91).

### Post Hoc Analyses

#### Self-Selected Use of Cessation Pharmacotherapy and e-Cigarettes as an Aid to Cessation

Over a third (188/444, 42.3% of adopters vs 438/1097, 39.9% of nonadopters; *P*=.43) of all participants reported using another form of nicotine replacement therapy (ie, nicotine patch, gum, or lozenge) or medications (ie, varenicline or bupropion) to help them quit smoking at 3 and 6 months. We found no evidence of an interaction between the use of pharmacotherapy and treatment arm assignment on the primary cessation outcome (*P*=.91). We also observed no difference in the rate of pharmacotherapy usage between adopters and nonadopters in either treatment arm (92/211, 43.6% adopters vs 227/554, 41% nonadopters; *P*=.66 in the iCanQuit arm and 107/254, 42.1% adopters vs 212/543, 39% nonadopters in the QuitGuide arm; *P*=.53).

Nearly half (219/465, 47.1%) of adopters reported using e-cigarettes as an aid to cessation. However, there was no difference in cigarette smoking cessation rates between those reporting they adopted e-cigarettes as an aid to cessation of cigarette smoking versus those who did not—in either the iCanQuit arm (19/85, 22% vs 22/105, 21%; *P*=.97; OR 0.99, 95% CI 0.47-2.06) or the QuitGuide arm (19/110, 17.3% vs 25/129, 19.4%; *P*=.69; OR 0.87, 95% CI 0.45-1.71).

#### Engagement With the App-Based Interventions

Table 3 provides information on participants’ engagement with their assigned app. In the iCanQuit arm, adopters had fewer logins to their assigned app than nonadopters, but the difference was not statistically significant (*P*=.19). Participants engaged less with the QuitGuide app overall, and in this treatment arm, there was no difference in app engagement between adopters compared with nonadopters (*P*=.27).

**Table 3:** Engagement with the app-based interventions between adopters and nonadopters for each treatment arm by 6 monthsa.

Treatment arm	Participants, n/N (%)	Nonadopters		Adopters			Incidence rate ratio (95% CI)		*P* value
		Mean (SD)	Median (IQR)	Mean (SD)	Median (IQR)				
iCanQuit	749/765 (97.9)	35.7 (68.2)	9 (3-39)	24.8 (51.4)	8 (2.5-20.5)	0.86 (0.68-1.08)		.20	
QuitGuide	790/797 (99.1)	9.1 (18.4)	5 (2-11)	10.0 (45.3)	5 (2-10)	1.10 (0.93-1.31)		.27	

^a^Negative binomial models include the following covariates: age, posttraumatic stress disorder positive screening, smoking first cigarette within 5 minutes of waking, and number of housemates who smoke.

#### Number of Quit Attempts

Adopters in both arms reported a significantly higher number of quit attempts compared with nonadopters (Table 4). In the iCanQuit arm, adopters reported a significantly higher number of quit attempts than nonadopters at 6 months (*P*=.02) but not at 12 months (*P*=.44). In the QuitGuide arm, adopters reported a significantly higher number of quit attempts than nonadopters at 6 months (*P*=.001) and 12 months (*P*<.001). Given the higher number of quit attempts among adopters in both treatment arms, we further evaluated whether the number of quit attempts at the 3- and 6-month follow-ups was associated with cigarette smoking cessation rates at 12 months. We found a very small negative association between 12-month cigarette smoking cessation and number of quit attempts by 3 months (*P*<.001; OR 0.999, 95% CI 0.999-0.999) and 6 months (*P*<.001; OR 0.999, 95% CI 0.999-0.9996).

**Table 4:** Association between e-cigarette use and number of quit attempts by treatment arm at each follow-up time point^a^.

Treatment arm		Participants, n/N (%)	Nonadopters		Adopters		Incidence rate ratio of point estimate (95% CI)	*P* value
			Mean (SD)	Median (IQR)	Mean (SD)	Median (IQR)		
**At 3 months^b^**								
	iCanQuit	750/765 (98)	5.6 (12.1)	2 (1-5)	6.4 (12.3)	3 (1-6)	1.15 (0.91-1.45)	.26
	QuitGuide	782/797 (98.1)	4.2 (8.0)	2 (0-4)	4.2 (6.9)	2 (1-4)	1.03 (0.83-1.27)	.80
**At 6 months^c^**								
	iCanQuit	747/765 (97.6)	8.8 (22.8)	3 (1-6)	10.8 (22.4)	3 (2-8)	1.32 (1.04-1.68)	.02
	QuitGuide	788/797 (98.9)	6.2 (14.2)	2 (1-5)	8.7 (19.9)	3 (2-6)	1.46 (1.17-1.81)	.001
**At 12 months^d^**								
	iCanQuit	711/765 (92.9)	13.2 (46.3)	3 (1-6)	12.0 (39.5)	3 (2-8)	1.11 (0.85-1.45)	.44
	QuitGuide	764/797 (95.6)	8.0 (23.6)	3 (1-6)	13.9 (40.6)	4 (2-9)	1.56 (1.25-1.94)	<.001

^a^All models were adjusted for factors used in stratified randomization including positive screening for depression, daily smoking frequency, education, and minority race or ethnicity backgrounds.

^b^Additional covariates are posttraumatic stress disorder positive screening and number of housemates who smoke.

^c^Additional covariates are posttraumatic stress disorder positive screening, smoking first cigarette within 5 minutes of waking, and number of housemates who smoke.

^d^Additional covariates are number of housemates who smoke.

#### Levels of Nicotine Dependence and Cigarette Smoking Cessation

Regarding the baseline difference in FTCD scores between adopters and nonadopters (6.0 vs 5.8; *P*=.01; Table 1) in our study, we did not find any evidence indicating that baseline levels of nicotine dependence, as measured by FTCD, were associated with combustible cigarette smoking cessation rates at 12 months (*P*=.69; OR 0.99, 95% CI 0.92-1.06). Similar observations emerged when assessing the cessation rates of cigarette smoking at 12 months, comparing individuals with low to moderate nicotine dependence (FTCD scores below 6.0) and those with high nicotine dependence (FTCD scores of 6.0 or higher) at baseline (147/594, 24.7% vs 228/894, 25.5%; *P*=.82; OR 1.03, 95% CI 0.80-1.34).

## Discussion

### Principal Findings

This study used data from an RCT to compare combustible cigarette smoking cessation rates between participants who used e-cigarettes on their own (ie, “adopters”) versus those who did not (ie, “nonadopters”). For the first time, this study provides empirical evidence that adults who adopted e-cigarettes while concurrently receiving an app-delivered cigarette smoking cessation intervention had either a lower or an unimproved likelihood of quitting combustible cigarette smoking—despite their strong motivation to quit. In the iCanQuit arm, 12-month combustible cigarette smoking cessation rates were significantly lower among adopters compared with nonadopters (41/193, 21.2% vs 184/527, 34.9%; *P*=.003). In contrast, in the QuitGuide arm, 12-month combustible cigarette smoking cessation rates did not differ between adopters and nonadopters (46/246, 18.7% vs 104/522, 19.9%; *P*=.64).

Our results are consistent with 2 previous prospective RCT studies for smoking cessation. Both studies suggested that e-cigarette use impeded cigarette smoking cessation among individuals concurrently receiving a behavioral intervention for smoking cessation [15,16]. The first study used data from an RCT (n=1040) among hospitalized patients who wanted to quit combustible cigarette smoking and received cessation behavioral counseling while at the hospital [15]. Patients were followed after discharge and assessed for cigarette smoking abstinence at the 6-month follow-up. The study found that those who started using e-cigarettes after discharge were significantly less likely than non–e-cigarette users to abstain from combustible cigarette smoking (quit rates, 10.1% for e-cigarette users vs 26.6% for non–e-cigarette users; *P*<.001). The second study used data from a large RCT (n=2637) among adults who smoked combustible cigarettes daily and were assigned to receive 12-month web-based behavioral interventions for cigarette smoking cessation [16]. The study found that those who used e-cigarettes were significantly less likely to quit combustible cigarettes than non–e-cigarette users at the 12-month follow-up (quit rates, 21.4% e-cigarette users vs 29.7% non–e-cigarette users; *P*=.006). In contrast to these studies, observational data suggest that e-cigarette use may facilitate smoking cessation under “free living conditions” [11-13]. Moreover, data from RCTs testing e-cigarettes as a potential treatment for smoking cessation suggest that e-cigarette use may help adults quit cigarette smoking [14,44,45]. The disparate results between observational studies, prospective studies, and RCTs of e-cigarettes may relate to discrepancies in how e-cigarettes are used in the real world versus in controlled research studies.

There are unclear reasons why e-cigarette adopters in this study had either a lower or an unimproved likelihood of quitting combustible cigarette smoking. Notably, we found no evidence that the self-selected use of cessation pharmacotherapy differed between adopters and nonadopters. We also examined whether other factors such as adopting e-cigarettes specifically to aid cessation had any impact on the 12-month cessation outcomes. Our findings revealed no significant associations in these regards. Regarding app engagement, however, we found that adopters in the iCanQuit arm used their assigned app less than nonadopters, albeit nonsignificantly, suggesting that adopting e-cigarettes may have been perceived as a method for quitting smoking—which could have thereby replaced app use. Given that engagement with an app-based intervention is a strong predictor of smoking cessation [46-51], it is possible that less engagement with the iCanQuit app contributed, at least in part, to the reduced odds of quitting smoking among adopters compared with nonadopters. However, in the QuitGuide arm, we found no difference in app use between adopters and nonadopters. Thus, further research is needed to understand why adopting e-cigarettes while concurrently receiving an app-based behavioral intervention to quit smoking may lead to reduced use of QuitGuide.

Finally, the number of quit attempts at 3-, 6-, and 12-month follow-ups between adopters and nonadopters was compared. Interestingly, we discovered that adopters had notably higher instances of quit attempts in comparison to nonadopters at the 6-month follow-up in the iCanQuit arm and at 6- and 12-month follow-ups in the QuitGuide arm. These results suggest that, in contrast to nonadopters, adopters encountered more difficulty with quitting cigarette smoking. Notably, these results could not be attributed to differences in levels of nicotine dependence between adopters and nonadopters in this study, which agrees with previous studies with mixed results [16,52-55]. These studies collectively underscore the complexities and uncertainties surrounding the direction of causality between nicotine dependence and e-cigarette use. Therefore, further randomized controlled research on the use of e-cigarettes for smoking cessation is necessary to draw more definitive conclusions.

### Strengths

This study has several strengths. First, this is the only known study to examine the impact of e-cigarette use among adults receiving an app-delivered behavioral intervention to help them quit smoking. Second, the original iCanQuit RCT enrolled a racially and geographically diverse large sample (n=2415) of adults from all 50 US states [21], increasing the generalizability of the findings to a broad range of adults who smoke across the country. Third, outcome data retention rates at the 12-month follow-up were high and did not differ between groups. Finally, the use of app-delivered interventions is also a notable strength, as it provides a convenient and accessible way for adults to access evidence-based cessation support.

### Limitations

There are also limitations of this study. As with any secondary analysis, the study was not specifically designed to examine the causal relationship between adopting e-cigarettes and cigarette smoking cessation; as such, a causal relationship may not be inferred from the associations found. Additionally, participants self-selected into using or not using e-cigarettes (as allocation was not randomized). This means that both measured and unmeasured confounding likely impact any associations seen. Nonetheless, we adjusted for any baseline characteristics that were significantly different between adopters and nonadopters and were associated with the cessation outcomes. Second, the study did not assess for type of e-cigarette used or its nicotine content. Third, self-reported data are subject to bias and inaccuracies, including in reporting smoking behavior and use of e-cigarettes. In this trial, the self-reported outcome was prespecified due to methodological issues with remote biochemical verification [56,57]. Although previous studies have demonstrated strong agreement between self-reported and biochemically verified smoking status [58,59], some have also shown significant discordance [60,61]. Therefore, the external validity of the self-reported smoking status in this trial is uncertain. However, because the trial was double-blinded, there is no compelling reason that the false reporting rate would be higher in one arm than the other.

### Conclusions

Adults who adopted e-cigarettes while simultaneously receiving an app-delivered smoking cessation intervention had either a lower or an unimproved likelihood of quitting cigarette smoking. This is concerning, given the increasing popularity of e-cigarette use. The study can inform future app-based cessation interventions and best practices to mitigate harm from cigarette smoking. Future studies and interventions may include education on the potential negative or unhelpful impact of using e-cigarettes alongside app-delivered behavioral interventions for cigarette smoking cessation.

## Abbreviations

ACT: acceptance and commitment therapy
FTCD: Fagerström Test for Cigarette Dependence
OR: odds ratio
PPA: point prevalence abstinence
PTSD: posttraumatic stress disorder
RCT: randomized controlled trial
USCPG: US Clinical Practice Guidelines
